# Purification and characterization of a novel, highly potent fibrinolytic enzyme from *Bacillus subtilis* DC27 screened from *Douchi*, a traditional Chinese fermented soybean food

**DOI:** 10.1038/s41598-019-45686-y

**Published:** 2019-06-25

**Authors:** Yuanliang Hu, Dan Yu, Zhaoting Wang, Jianjun Hou, Rohit Tyagi, Yunxiang Liang, Yongmei Hu

**Affiliations:** 1 State Key Laboratory of Agricultural Microbiology, College of Life Science and Technology, Huazhong Agricultural University, Wuhan, 430070 China; 2 Hubei Key Laboratory of Edible Wild Plants Conservation& Utilization, College of Life Sciences, Hubei Normal University, Huangshi, 435002 China; 3 Hubei Collaborative Innovation Center for Industrial Fermentation, Wuhan, 430068 China

**Keywords:** Proteases, Bacterial genomics, RNA sequencing, Applied microbiology

## Abstract

The highly fibrinolytic enzyme-producing bacterium was identified as *Bacillus subtilis* DC27 and isolated from *Douchi*, a traditional fermented soybean food. The DFE27 enzyme was purified from the fermentation broth of *B*. *subtilis* DC27 by using UNOsphere Q column chromatography, Sephadex G-75 gel filtration, and high-performance liquid chromatography. It was 29 kDa in molecular mass and showed the optimal reaction temperature and pH value of 45 °C and 7.0, respectively, with a stable fibrinolytic activity below 50 °C and within the pH range of 6.0 to 10.0. DFE27 was identified as a serine protease due to its complete inhibition by phenylmethysulfony fluoride. The first 24 amino acid residues of the N-terminal sequence of the enzyme were AQSVPYGVSQIKAPALHSQGFTGS. The enzyme displayed the highest specificity toward the substrate D-Val-Leu-Lys-pNA for plasmin and it could not only directly degrade but also hydrolyze fibrin by activating plasminogen into plasmin. Overall, the DFE27 enzyme was obviously different from other known fibrinolytic enzymes in the optimum substrate specificity or fibrinolytic action mode, suggesting that it is a novel fibrinolytic enzyme and may have potential applications in the treatment and prevention of thrombosis.

## Introduction

Currently, cardiovascular diseases (CVDs) are dominant causes of death worldwide for people with hypertension, myocardial infarct, coronary heart disease, or stenocardia^1^. Consistent with the estimation of World Health Organization(WHO), CVDs caused 15.6 million deaths in 2010 worldwide and are expected to cause more than 20 million deaths per year by 2020^2,3^. In the various types of CVDs, thrombosis is among the most commonly occurring diseases of modern life and could be responsible for the increasing number of deaths^4,5^.

The fibrinolytic system can not only dissolve fibrin clots resulting from fibrinogen through activated thrombin (EC 3.4.21.5), but also provide a channel for blood flow at the site of vascular injury as hemostatic response^6,7^. Additionally, the plasmin also hydrolyzes fibrin clots at the site of injury (EC 3.4.21.7) to prevent thrombosis^2^. The clots which are not hydrolyzed lead to the occurrence of thrombosis under unstable conditions like myocardial infraction or disorders such as hypertension^8^.

The most effective drugs for treating thrombosis are fibrinolytic enzymes, which can be divided into two types according to their action mode: plasminogen activators (PAs), including tissue plasminogen activator (t-PA), streptokinase (SK, 3.4.99.22) and urokinase (UK, EC 3.4.21.31), which can lyse fibrin through the formation of plasmin from plasminogen^9,10^, and plasmin-like fibrinolytic enzymes, which can degrade the fibrin directly^2^. Despite extensive research about them and their wide use as thrombolytic agents, these PAs are high in price and low in specificity, prompting the researchers to explore for safer and cheaper resources. Microorganisms contribute to the production and use of the fibrinolytic enzymes since ancient times, and researchers have paid increasing attention to the production of fibrinolytic enzymes by microorganisms, particularly those from the food sources. During past decades, several fibrinolytic enzymes have been recognized from different microorganisms, especially the genus *Bacillus*^5,11–14^.

Recent studies have shown that various potent fibrinolytic enzymes can be produced from different kinds of traditional fermented foods, for example, Japanese *natto*^15^, Chinese fermented paste^16^, fermented red bean^17^, *Douchi*^4,5,18,19^, *Doenjang*^13,20^, *cheonggukjan*g^21^, Indonesian *tempeh*^12^, and Egyptian *Kishk*^14^. It has been confirmed that Nattokinase (NK) can directly lyse thrombi *in vivo* and its oral administration can improve plasma fibrinolytic activity as well as t-PA production^22–24^. Similar fibrinolytic enzymes from *Douchi* were also confirmed to degrade fibrin directly and efficiently in *vitro* and in *vivo*^5,25^. In this article, a novel enzyme with potential fibrinolytic activity was isolated, purified and characterized from the bacterial strain *B*. *subtilis* DC27 in *Douchi*, a fermented soybean food popular in China since ancient times.

## Materials and Methods

### Isolation and identification of bacterial strain

The dry *Douchi* samples obtained from Jiatai Co., Ltd., Chongqing, China were soaked in sterile saline for 20 min at 37 °C, treated at 80 °C for 10 min, and isolated by serial dilution in sterile saline. After centrifugation at 4,000 rpm for 5 min, the culture supernatants were cultured on the nutrient agar broth containing casein at 37 °C for 18 h, followed by collecting the single colonies and culture in Luria-Bertani medium at 37 °C for 72 h under shaking.

The physiological and biochemical characteristics of the isolates were identified as described in the Bergey’s Brochure for identification of Bacteriology. The extraction of total genomic DNA was performed using the Genomic Tip-100 kit (Qiagen, Hilden, Germany) for 16 S rDNA identification, and the gene was PCR amplified using primers of 5′-AGAGTTTGATCCTGGCTCAG-3′ and 5′-ACGGCTACCTTGTTACGACT-3′ ^26^. The 16S rDNA sequence homology analysis was performed using the basic local alignment search tool (BLAST) at the website of NCBI (http://www.ncbi.nlm.nih.gov).

### Fibrinolytic activity assay

A minorly modified fibrin plate method was used to determine the fibrinolytic activity^27^. Specifically, 0.15% fibrinogen (bovine; NICPBP, Beijing, China) was mixed with 10 ml of 60 mM sodium phosphate buffer (pH 7.4), and 1 ml of thrombin (bovine; NICPBP, Beijing, China) (5 U ml^−1^) was dissolved in 10 ml of 1.5% agarose gel. Next, the two solutions were well mixed and poured into the plates, followed by treatment at 37 °C for 30 min to allow the formation of fibrin clots, and punching nine holes on fibrin coagulum by a duchenne tubular (diameter 2 mm). After heating at 80 °C for 30 min to inactivate the plasminogen (bovine; NICPBP, Beijing, China), the plates were supplemented with 10 μl of enzyme solutions through the 9 punched holes and then incubated at 37 °C for 18 h. The fibrinolytic enzyme activity was measured using the diameter of transparent zone on the fibrin plate based on urokinase (60 000 IU/mg; NICPBP, Beijing, China) standard solutions. The activity of each milligram protein is defined as an enzyme activity unit (IU/mg). With bovine serum albumin used as a criterion, the protein concentration was measured with the bicinchoninic acid protein assay reagent kit (Sigma, Beijing, China).

### Enzyme purification

The DFE27 enzyme was purified successively at 4 °C by UNOsphere Q column chromatography, Sephadex G-75 gel filtration, and high-performance liquid chromatography (HPLC). Firstly, the proteins of 5 L medium supernatant were separated by 40–70% (NH_4_)_2_SO_4_ precipitation, followed by centrifugation at 10,000 rpm for 15 min to acquire target proteins, dissolution of the protein pellets in 20 mM Tris-HCl buffer (pH 8.8) and dialysis against the same buffer overnight. Secondly, the dialyzed enzyme was concentrated and then loaded on a UNOsphere Q anion exchange column, followed by elution at a rate of 1.0 ml/min with 20 mM Tris-HCl buffer (pH 8.8) containing 1 M of NaCl and measuring the enzyme activity and protein concentration separately from the collected elution peaks. Next, the highly active solution was passed through a Sephadex G-75 (Pharmacia, Shanghai, China) column (1.5 cm × 25 cm) previously balanced with 20 mM Tris-HCl buffer (pH 7.0) at a flow speed of 0.2 ml/min. Meanwhile, the elution peaks were collected separately for measuring the protein concentration and enzyme activity, and the portion with highest enzyme activity was further concentrated and freeze-dried. Lastly, the freeze-dried portion was dissolved with 200 mM Na_2_HPO_4_ solution, and HPLC analyses were performed with a GF-250 column (9.4 mm × 250 mm) on an Agilent 1200 series instrument by using 200 mM Na_2_HPO_4_ solution at 1 ml/min, 23 °C and 280 nm. The absorption peaks were collected and the purity of the freeze-dried products was measured.

### SDS-PAGE

To evaluate the purity and molecular mass of the proteins, SDS-PAGE was done at room temperature on a Modular Mini-Protein II Electrophoresis System (Bio-Rad, Hercules, USA). Specifically, the proteins were electrophoresed in a 5% stacking gel and 12% resolving gel of polyacrylamide, followed by staining with 0.25% Coomassie Blue R-250 (Sigma, Beijing, China).

### Zymography

To perform fibrin zymography as described in the literature^28^, 0.12% (w/v) fibrinogen and 100 μl thrombin (10 IU/ml) were used to prepare a 12% polyacrylamide gel. After electrophoresis at 12 mA and 4 °C, the gel was incubated for 30 min at room temperature in 50 mM pH 7.0 Tris-HCl containing 2.5% (v/v) Triton X-100, followed by washing in distilled water for 30 min and incubation at 37 °C for 12 h in the zymogram reaction buffer consisting of 0.02% (w/v) NaN_3_ and 30 mM pH 7.0 Tris-HCl^29^. Finally, after staining with Coomassie blue R-250 for 2 h, the gel was destained, and the non-staining regions of the fibrin gel were defined as the active bands.

### Effects of pH and temperature on DFE27 activity

To measure the effects of pH on the fibrinolytic activity, the purified enzyme solution was adjusted to the fibrinolytic activity of 50 IU/ml at 37 °C and maintained for 30 min in the following different buffer solutions (0.1 M): HAc-NaAc buffer (pH 4.0, 5.0), Tris-HCl buffer (pH 8.0, 9.0), phosphate buffer (pH 6.0, 7.0), and glycine-NaOH buffer (pH 10.0, 11.0). To measure pH stability, the enzyme was incubated in the buffers separately from pH 5.0 to 10.0 at 37 °C for 0, 10, 30, 60 and 90 min. After readjusting the pH to 7.5, the residual fibrinolytic activity was determined. The effects of temperature on the fibrinolytic activity were determined by incubating the enzyme at different temperatures for 30 min and then measuring the residual activity (purified fibrinolytic enzyme solution in 20 mM pH 7.5 Tris-HCl buffer, 50 IU/ml). The thermal stability was investigated by assaying the residual fibrinolytic activity in pH 7.5 Tris-HCl buffer at 37, 45, 50 and 60 °C for 0, 30, 60, 90, and 120 min.

### Effect of inhibitors and metal ions on DFE27 activity

The fibrinolytic enzyme type was determined by evaluating the effects of multifarious inhibitors on the fibrinolytic activity. Briefly, the purified enzyme solution (50 IU/ml) was pre-incubated at 37 °C for 30 min separately with each of the following inhibitors: 5.0 mM serine protease inhibitor phenylmethanesulfonyl fluoride (PMSF; Sigma, Beijing, China), 5.0 mM metalloprotease inhibitor ethylene diamine tetraacetic acid (EDTA; Sigma, Beijing, China), 10 µM aspartic protease inhibitor pepstatin A (Sigma, Beijing, China), 1.0 mM cysteine protease inhibitor trans-epoxysuccinyl-L-leucylamido-(4-guanidino) butane (E64; Sigma, Beijing, China), and 1.0 mM aminopeptidase inhibitor bestatin (Sigma, Beijing, China). To measure the effects of metal ions on the activity, the fibrinolytic enzyme (50 IU/ml) was pre-incubated separately with different metal ions (final concentration 5 mM) at pH 7.5 and 37 °C for 30 min. The enzyme solution without added ions was used as a control.

### Substrate specificity of DFE27

The DFE27 specificity was evaluated by using the synthetic substrates of N-succinyl-Ala-Ala-Pro-Phe-p-nitroanilide (*p*NA) (characteristic substrate for subtilisin or chymotrypsin; Sigma, Beijing, China), D-Val-Leu-Lys-*p*NA (characteristic substrate for plasmin; Sigma, Beijing, China), and D-Val-leu-Arg-*p*NA (characteristic substrate for kallilrein; Sigma, Beijing, China). The reaction mixture contained 8 μl of 1 mM synthetic substrate, 10 μl of enzyme solution and 20 μl of 100 mM pH 7.0 Tris-HCl buffer. After incubating the mixture at 37 °C for 30 min, the absorbance of released *p*NA at 405 nm was measured with a spectrophotometer to determine the amidolytic activity.

### PCR, sequencing and sequence analysis

DNA fragment was PCR amplified with the TIANamp Bacteria DNA Kit (Tiangen, Shanghai, China) using the primers 5′-CTGAATCAGGGAAATCAAACGG-3′ and 5′-GTCGTTACTGAGCTGCCGCCTGTAC-3′ for forward and reverse reactions^30^, respectively. The 50 µl of reaction mixture consisted of 5 µl of 10x PCR buffer, 0.5 µl of Taq polymerase, 1 µl of genomic DNA, 4 µl of 2.5 mM dNTP, 37.5 µl of ddH_2_O and 1 µl of each of primers. The PCR was performed under the conditions of 1 cycle at 94 °C for 3 min, 30 cycles (at 94 °C and 55 °C for 1 min and 72 °C for 2.5 min), and a final cycle at 72 °C for 10 min. After purifying and sequencing the PCR product, the nucleotide acid sequence of this enzyme was converted into amino acid sequence at the website http://web.expasy.org/translate/. Multiple sequence alignments of DFE27 and other enzymes were performed using GeneDoc software.

### Statistical analysis

All the data were presented as the mean ± SD and subjected to analysis of variance and *F* test using SPSS 11.0 statistical software (SPSS, Inc. Chicago, IL, USA).

## Results

### Strain isolation and identification

A total of 156 strains showing fibrinolytic activity were isolated from 7 samples, and the isolate DC27 with the strongest fibrin-degrading activity was chosen for further study. The results showed that the strain DC27 was Gram positive, rod shaped and endospore forming (more details are shown in Supplemental Table S1), with a typical characteristic of *B*. *subtilis*. The 16 S rDNA sequence obtained from it (1461 bp; NCBI no. MK779713) had 99% sequence similarity to that of *B*. *subtilis* DSM 10 (NCBI no. NR_027552.1). The strain was identified as *B*. *subtilis* and designated as *B*. *subtilis* DC27 which was deposited at the China Center for Type Culture Collection (CCTCC, M2015573).

### Optimization of fermentation conditions

In order to obtain more enzymes, the medium components and culture conditions were optimized. Results showed that soluble corn starch and soybean peptone were the optimal carbon and nitrogen sources, respectively (Supplemental Table S2). The optimal conditions consisted of medium volume 40 ml/250 ml, inoculum size 2%, initial pH 8.0, and incubation temperature 32 °C (Supplemental Fig. S1).

### Purification of DFE27

**Figure 1:**
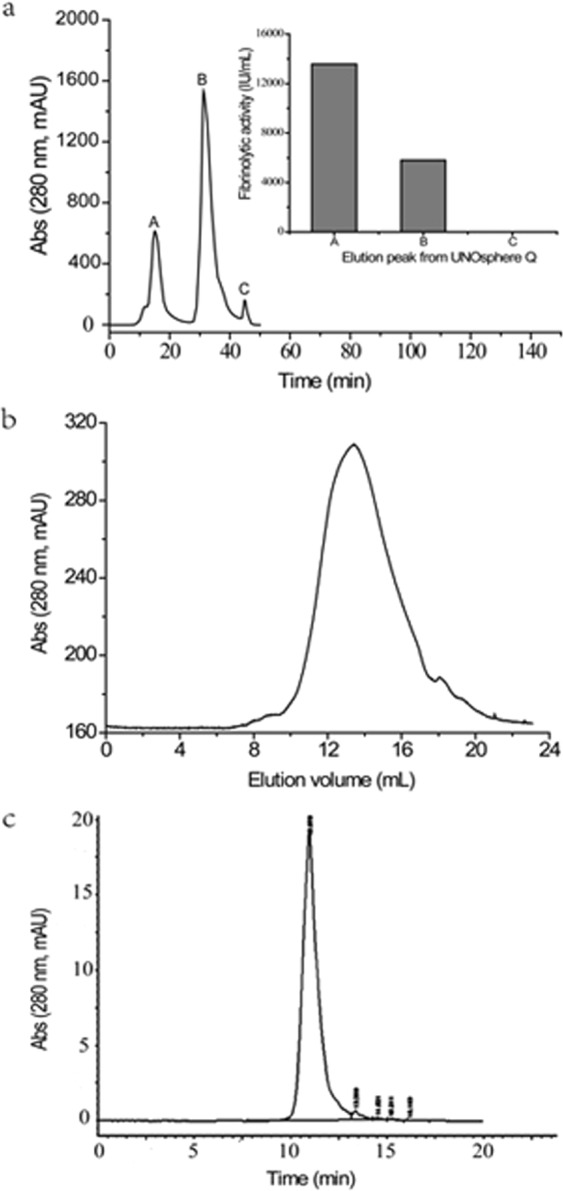
The purification of DFE27 by chromatography. (**a**) Purification of DFE27 using UNOsphere Q anion-exchange column for stepwise gradient elution with 20 mM Tris-HCl buffer (pH 8.8) containing 1 M of NaCl at a flow rate of 1.0 ml/min and 4 °C; (**b**) Further purification of DFE27 by Sephadex G-75 column chromatography eluted with 20 mM Tris-HCl buffer (pH 7.0) at a flow rate of 0.2 ml/min and 4 °C; (**c**) HPLC purification of DFE27 using 200 mM Na_2_HPO_4_ solution as mobile phase at a flow rate of 1 ml/min, a column temperature of 23 °C and a detection wavelength of 278 nm.

The chromatograms are shown in Fig. 1, and the protease yield and purity in each purification step are presented in Table 1. In Fig. 1a, proteins in peak A and B exhibited fibrinolytic activities, with the highest fibrinolytic activity in peak A, thus proteins in peak A were collected and purified by Sephadex G-75. The elution of a sephadex G-75 column resulted in a major single elution peak based on the protein size (Fig. 1b). The freeze-dried fraction from 65 ± 2 min of elution time in sephadex G-75 gel filtration was further purified by HPLC (Fig. 1c), and a sharp single peak occurred at around 10.978 min. After purification by UNOsphere Q, Sephadex G-75 and HPLC, DFE27 was 69.1-fold purified with a yield of 12.73% relative to that of the crude enzyme (Table 1). The specific activity was estimated to be 11274.4 IU/mg (urokinase activity units/mg of protein) for the ultimate enzyme.

**Table 1:** Purification of DFE27 from *B*.*subtilis* DC27.

Purification step	Total protein (mg)	Total activity (IU)	Specific activity (IU/mg)	Yield (%)	Purification fold
Medium supernatant	6929.82	1130098	163.1	100	1
(NH_4_)_2_SO_4_	1387.10	675288	486.8	59.75	3.0
UNOsphere Q	61.42	361287	5882.2	31.97	36.1
Sepharose G-75	15.56	167493	10764.3	14.82	66.0
HPLC	12.76	143861	11274.4	12.73	69.1

### Molecular mass of DFE27

**Figure 2:**
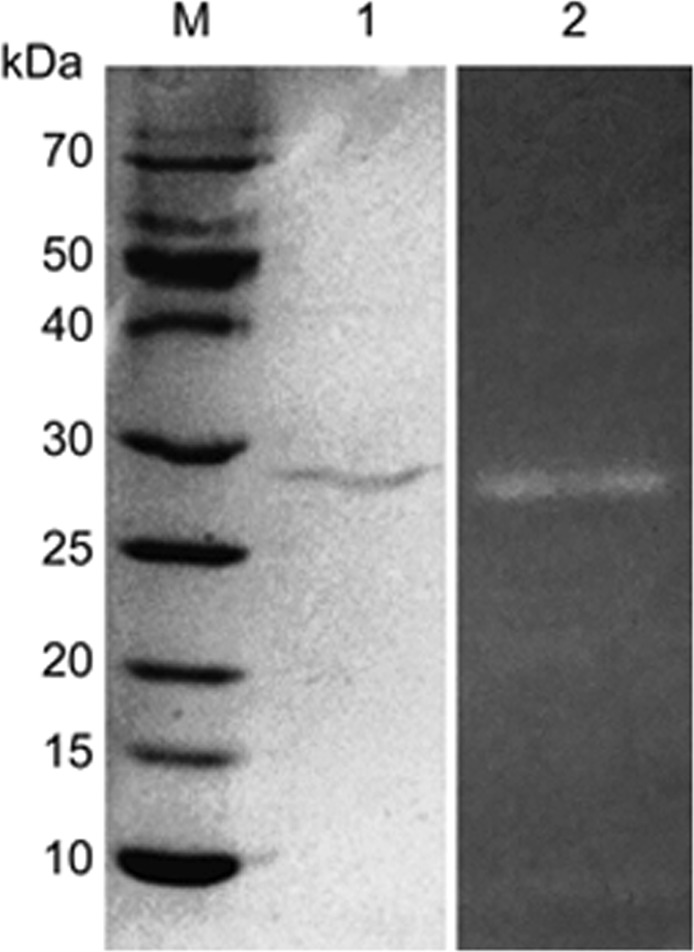
The purity and molecular mass of the purified DFE27 enzyme as determined by SDS-PAGE and fibrin zymography. M, molecular mass standards; 1, The purified DFE27; 2, Fibrin zymography of the purified DFE27.

The molecular mass (Mr) of purified DFE27 was determined to be 29 kDa by SDS-PAGE (Fig. 2). The clear band indicated that the protein was purified to homogeneity by HPLC and it contained a single subunit or several subunits with the identical Mr of 29 kDa (Lane 1). The enzyme assay also revealed that the purified DFE27 had a molecular mass of about 29 kDa, with a very clear hydrolyzed band of approximately 29 kDa detected on the fibrin zymography (Lane 2).

### Optimum pH, temperature and stability of DFE27

**Figure 3:**
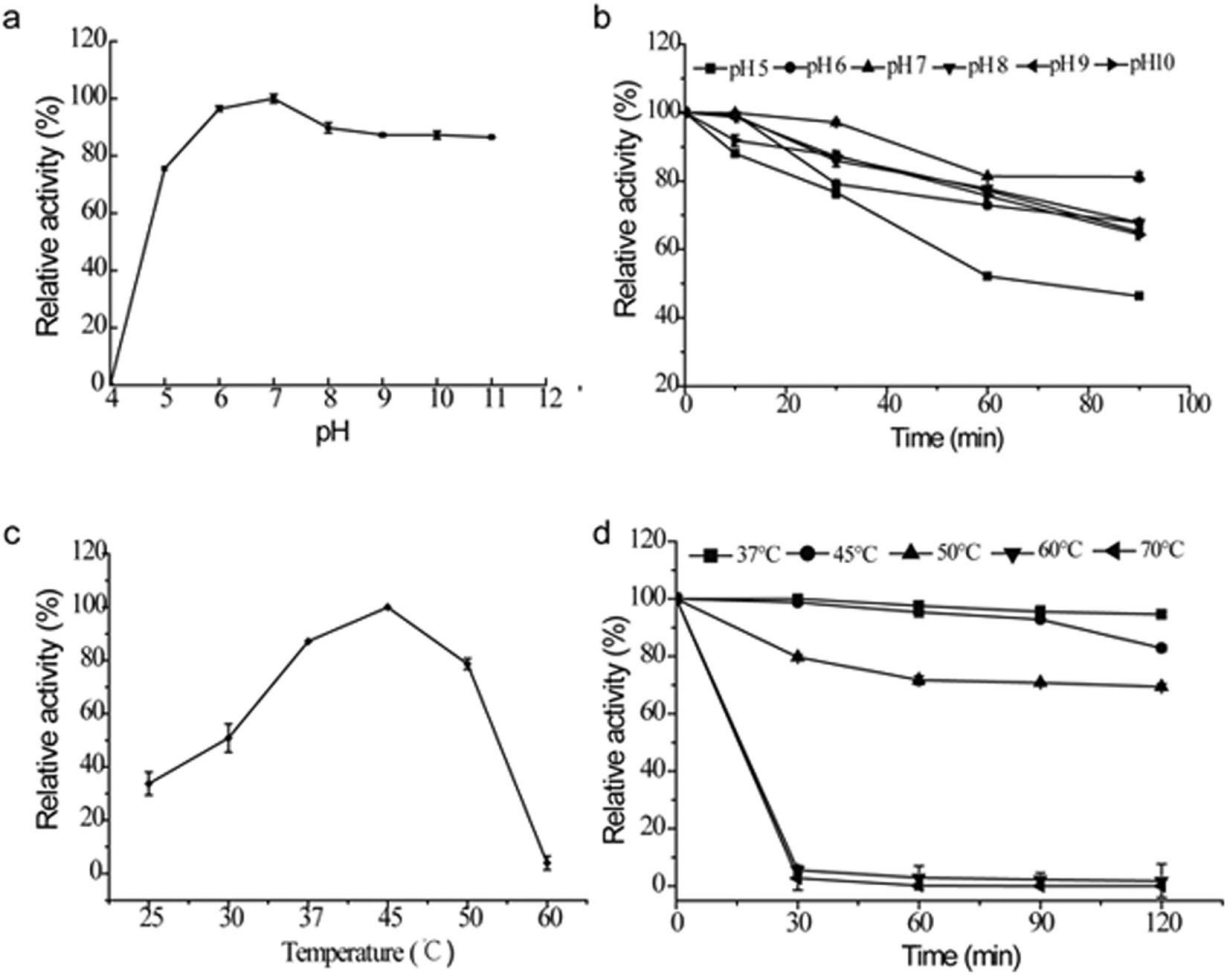
Effects of pH and temperature on fibrinolytic activity and stability of DFE27. (**a**) Optimal pH value was obtained by incubating the DFE27 enzyme in buffer solutions with a different pH value at 37 °C for 30 min to determine the fibrinolytic activity; (**b**) pH stability was determined by incubating the enzyme in different buffers at pH 5.0 to 10.0 and measuring the residual fibrinolytic activity at 37 °C after adjusting to pH 7.5; (**c**) Optimal temperature was determined by incubating the enzyme at different temperatures at pH 7.5 for 30 min to determine the activity; (**d**) Thermostability was determined by incubating the enzyme in pH 7.5 Tris-HCl buffer at 37, 45, 50 and 60 °C for 0, 30, 60, 90, and 120 min and measuring the residual fibrinolytic activity. Values are means ± SD from three independent experiments.

The DFE27 showed the highest activity at pH 7.0 (Fig. 3a), and was relatively stable at pH 6.0 to 10.0 and 37 °C for 90 min, but the enzyme stability abruptly decreased at pH 5.0 (Fig. 3b). Moreover, DFE27 showed the highest activity at 45 °C (Fig. 3c). The purified enzyme remained stable from 37 °C to 50 °C, unstable at a temperature above 60 °C, and exhibited almost no activity after treatment at 60 °C and 70 °C for 30 min (Fig. 3d).

### Effects of inhibitors and metal ions on DFE27 activity

The effects of a number of known protease inhibitors and 7 metal ions on DFE27 activity are presented in Table 2. DFE27 was completely inhibited by PMSF, suggesting it is a serine protease. However, the enzyme activity was increased by 122.02% and 121.75% by Ca^2+^, and K^+^, respectively. Furthermore, the enzyme activity was slightly influenced by Mn^2+^, Zn^2+^ and Mg^2+^, but significantly inhibited by Cu^2+^ and Fe^2+^ with a decrease of more than 81.78% and 39.07% respectively when compared with the control.

**Table 2:** Effects of protease inhibitors and metal ions on DFE27 activity.

Inhibitor/metal ion	Concentration	Relative activity (%)
None	—	100.00 ± 0.26
PMSF	5 mM	0.00 ± 0.00
EDTA	5 mM	85.99 ± 2.11
Pepstatin A	10 µM	88.91 ± 1.38
E64	1 mM	94.65 ± 0.90
Bestatin	1 mM	91.97 ± 0.00
Mn^2+^	5 mM	104.79 ± 0.30
Zn^2+^	5 mM	97.93 ± 9.06
Cu^2+^	5 mM	18.22 ± 0.91
Ca^2+^	5 mM	122.02 ± 5.71
Fe^2+^	5 mM	61.93 ± 4.28
K^+^	5 mM	121.75 ± 1.43
Mg^2+^	5 mM	90.00 ± 0.43

### Amidolytic activity of DFE27

Several synthetic substrates were used to evaluate the amidolytic activity of the purified DFE27 (Table 3). D-Val-Leu-Lys-*p*NA (for plasmin) was the most sensitive substrate for DFE27, with lesser effects from N-Suc-Ala-Ala-Pro-Phe-*p*NA (for chymotrypsin or subtilisin) and D-Val-Leu-Arg-*p*NA (kallikrein).

**Table 3:** Synthetic substrate specificity of DFE27.

Synthetic substrate (1 mM)	Substrate hydrolysis (mmol·min^−1^·ml^−1^)	Target enzymes
N-Suc-Ala-Ala-Pro-Phe-*p*NA	12.527	Subtilisin or chymotrypsin
D-Val-Leu-Lys-*p*NA	225.291	Plasmin, plasminogen
D-Val-Leu-Arg-*p*NA	47.656	Kallikrein

### Fibrinolytic action mode of DFE27

**Figure 4:**
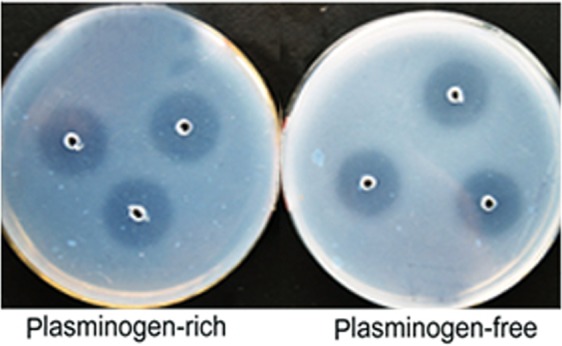
The fibrinolytic action mode. The fibrinolytic activity of DFE27 was detected on the plasminogen-rich and -free plates. The fibrin plate without heat treatment was considered as plasminogen-rich plate, and the fibrin plate with the 80 °C heat treatment for 30 min to inactivate plasminogen was defined as plasminogen-free plate.

Fibrin plate, without heat treatment, was viewed as rich in plasminogen, because commercially purchased fibrinogen usually contains only a small amount of plasminogen, while the fibrin plate, with a 30 min heat treatment at 80 °C to inactivate plasminogen, is defined as free from plasminogen. As shown in Fig. 4, clear hydrolyzed zones were formed in both the plasminogen-free and -rich plates. The fibrinolytic activity of DFE27 in the plasminogen-free plate was approximately 90% of the activity in the plasminogen-rich plate (Fig. 4), indicating that the purified DFE27 can not only directly hydrolyze fibrin, but also convert plasminogen to plasmin.

### Amino acid sequence alignment of DFE27 with other serine proteases

**Figure 5:**
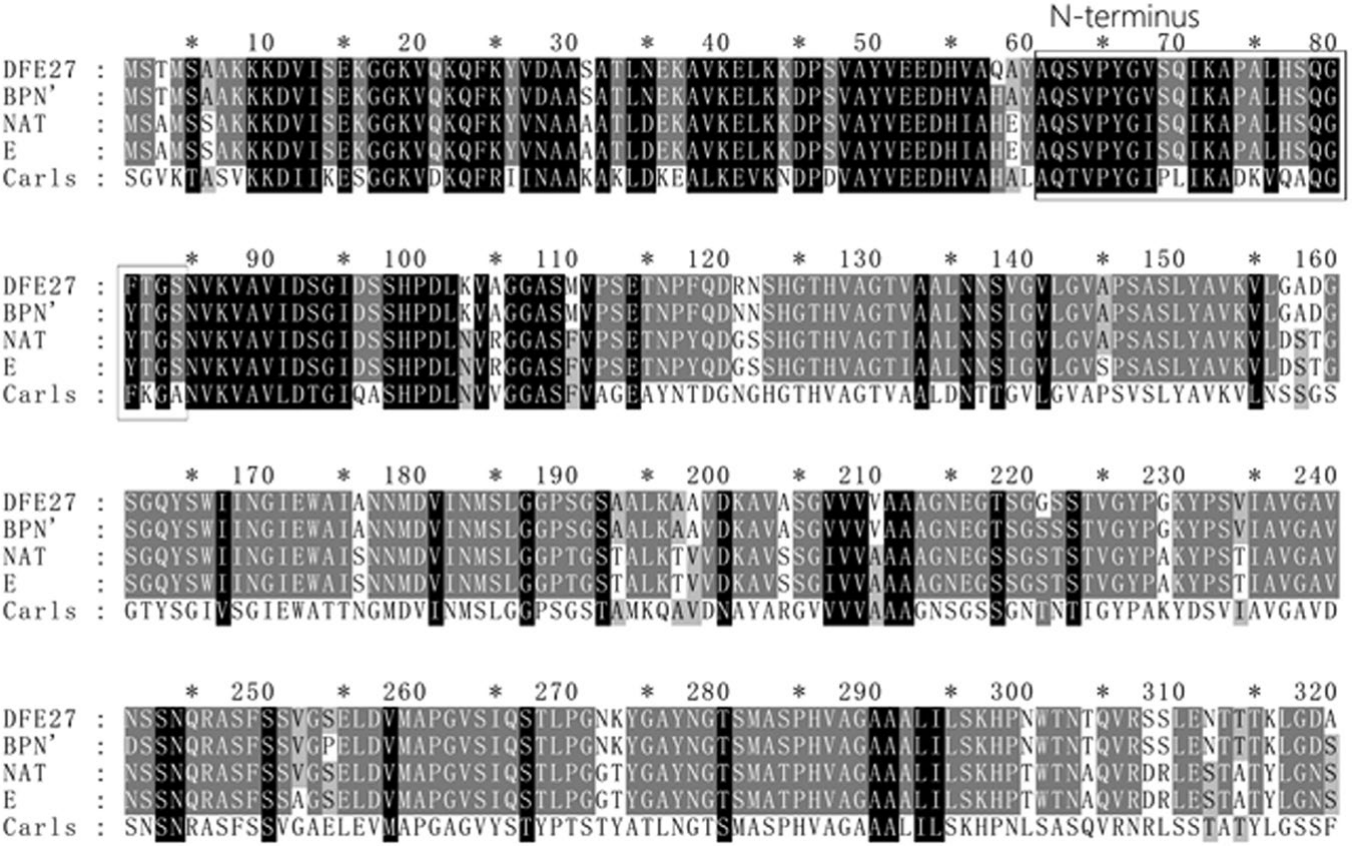
Multiple sequence alignment of DFE27 and other serine proteases. DFE27 from *B*. *subtilis* DC27, present study; BPN’ from *B*. *amyloliquefaciens*, K02496.1; NAT from *B*. *subtilis*, S51909.1; E from *B*. *subtilis*, K01988.1; Carls from *B*. *licheniformis*, X03341.1. The closed rectangle shows the N-terminal sequence of the first 24 amino acids.

Multiple sequence alignment of DFE27 and other serine proteases was performed and the results are shown in Fig. 5. Sequence analysis and alignment results showed that DFE27 had high similarity to subtilisin BPN’ (K02496.1) with 98% identity, and the partial sequence showed 86%, 85%, 67% identity to that of NAT (S51909.1), E (K01988.1) and Carls (X03341.1), respectively. At the N-terminus of the enzyme, the initial 24 amino acid residues were found to be AQSVPYGVSQIKAPALHSQGFTGS, which was different from that of BPN’, NAT, E and Carls.

### Comparison of DFE27 with other fibrinolytic enzymes

Comparative analysis of DFE27 with other fibrinolytic enzymes was shown in Table 4, revealed that DFE27 displayed unique substrate specificity and fibrinolytic action mode, which are different from other fibrinolytic enzymes originated from fermented foods. It is obvious that the sequence of DFE27 and DFE showed high homology, but the originated microorganism, fibrinolytic action mode and substrate specificity are extremely different. Furthermore, the initial 24-amino-acid residues were different from that of BsfA^28^, subtilisin QK-2^29^ and subtilisin NAT (NK)^30^, and the amino acid sequence of DFE27 showed only 85%, 84%, 86% similarity with BsfA, QK-2 and NAT, which are from fermented foods, respectively.

**Table 4:** Comparison of DFE27 and other fibrinolytic enzymes.

Fibrinolytic enzyme	Source	Microorganism	Molecular weight (kDa)	Substrate specificity	Fibrinolytic action mode	Amino acid similarity (%)	NCBI Sequence No.	Reference
DFE27	*Douchi*	*B*. *subtilis* DC27	29	D-Val-Leu-Lys-pNA	Direct and indirect	100	MG860912.1	Present study
Subtilisin DFE	*Douchi*	*B*. *amyloliquefacien*s DC-4	28	Suc-Ala-Ala-Pro-Phe-pNA	Direct	99	AAZ66858.1	Peng *et al*. 2003
BsfA	*Kimchi*	*B*. *subtilis* ZA400	28.4	ND	ND	85	JN392072.1	Ahn *et al*. 2015
Subtilisin QK-2	Fermented soybean	*B*. *subtilis* QK02	28	Suc-Ala-Ala-Pro-Phe-pNA	Direct	84	AMM02745.1	Ko *et al*. 2004
NAT	*Natto*	*B*. *subtilis* (*natto*)	30	Suc-Ala-Ala-Pro-Phe-pNA	Direct	86	S51909.1	Nakamura *et al*. 1992

ND, the result was not determined.

## Discussion

Thrombolytic therapies with plasminogen activators (t-PA, SK and UK) have high potential in treating thrombosis, facilitating the use of plasminogen activators in medicine today, but their high prices and side effects limit their wide application. Microbial fibrinolytic enzymes, isolated from food-grade microorganisms, are more in demand than typical thrombolytic agents. Nattokinase (NK) isolated from *natto* in 1987 is one of the most important discoveries in fibrinolytic enzymes, and a daily oral administration of as low as 50 g of *natto* or 2,000 fibrin units may help prevent the conditions leading to thrombosis^31^. Moreover, NK can maintain its activity for 2–12 h, whereas a fibrinolytic injection can only retain its efficiency for 4–20 min. Currently, NK is often employed as a fibrinolytic enzyme in hospitals^32^. Japan has the highest life expectancy around the globe, probably partially due to the increased use of fermented soybean food *natto*^2^.

*Douchi*, another fermented soybean food in China, is as ancient as *natto* and was also found to contain fibrinolytic enzymes formed by the bacteria harbored in it. In view of their impressive thrombolytic efficacy and safety, these microbial fibrinolytic enzymes may be excellent candidates for thrombolytic therapies. In this study, a highly potential 29 kDa fibrinolytic protease ‘DFE27’, as estimated by both SDS-PAGE and fibrin zymography, was identified from *B*. *subtillis* isolated from *Douchi*. Most fibrinolytic enzymes from food have a molecular mass between 28 and 30 kDa, and the molecular mass of DFE27 (29 kDa) was similar to that of Subtilisin DFE (28 kDa)^18^, BsfA (28.4 kDa)^33^, Subtilisin QK-2 (28 kDa)^34^ and NAT(30 kDa)^35^. Furthermore, the activity of DFE27 was obviously inhibited by PMSF, proving that it is a serine protease. To date, most known fibrinolytic enzymes such as nattokinase and DFE are serine proteases^18,31^.

The results from chromogenic substrates showed that DFE27 can perform the function of t-PA in hydrolyzing the synthetic substrate D-Val-Leu-Lys-*p*NA for plasmin, suggesting that DFE27 is a serine protease similar to plasmin and can degrade plasminogen in vivo as a potential fibrinolytic agent. Similar characteristics were found in serine proteases which are isolated from *Pleurotus eryngii*, *Periserrula leucophryna*, *Porcellio scaber* Latreille, *Cordyceps militaris* and *Asterina pectinifera*, respectively^36–40^. The optimum substrate of DFE27 is also similar to that of fibrinolytic enzymes obtained from fermented foods, such as *Natto* and Chungkook-jang^31,41^, but different from that of fibrinolytic enzymes of *Douchi*^4,18^.

DFE27 could also hydrolyze the fibrin independently as indicated by a clear band of 29 kDa on fibrin zymogram as well as its high fibrinolytic activity on both plasminogen-rich and -free plates, demonstrating its strong plasmin-like activity to lyse fibrin and fibrinogen. Interestingly, DFE27 was found to have the ability to activate plasminogen into plasmin, as evidenced by its performance in stimulating fibrinolytic activity on the plasminogen-rich fibrin plate and cleaving fibrin and fibrinogen as a direct acting fibrinolytic agent. This effect is in good agreement with that of fibrinolytic enzymes from *Pleurotus eryngii*^36^, *Paecilomyces tenuipes*^42^, *Streptomyces* sp.CS684^43^, and *Asterina pectinifera*^40^. Collectively, the bifunctional DFE27 enzyme is obviously superior to all the other known fibrinolytic enzymes in thrombolysis.

It is worth noting that the enzyme activity reached 5145.53 ± 52.00 IU/ml using a 50 L fermenter under the optimal culture conditions (data not shown). This is probably the highest yield level of *Douchi* fibrinolytic enzyme in the strains reported in the literature so far. Recently, statistical methods such as response surface methodology and central composite design have been effectively and frequently used to optimize the process parameters for enhancing the enzyme production^44–46^. Meanwhile, several genetically engineered strains, such as *B*. *subtilis*, *Escherichia coli*, and *Pichia*, are constructed for high production of enzyme. Therefore, *B*. *subtilis* DC27 has great application prospect for industrial production and further research due to its excellent characteristics for high yield.

DFE27 was classified as a serine protease based on its activity toward protease inhibitors and shared high similarity with subtilisin DFE (99%) and subtilisin BPN’(97.5%), two serine proteases originated from *B*. *amyloliquefaciens*. However, DFE27 showed significant differences from them in terms of strain source, optimum substrate specificity, fibrinolytic action mode or N-terminal sequence. For example, the N-terminal 24-amino-acids sequence of DFE27 was different from that of other fibrinolytic enzymes originated from other Asian fermented foods, such as *natto*^31,35^, fermented soybean^34^, doen-jang^28^, Chungkook-jang^41^ and kimchi^33^. In addition, even though showing identical action mode, the DFE27 exhibited different N-terminal sequence or substrate hydrolysis characteristic with other *Bacillus* fibrinolytic enzyme originated from *Douchi*, such as fibrinolytic enzyme FS33 and another enzyme from *Bacillus* sp. nov. SK006 screened from *shrimp paste*^4,47^.

DFE27 showed only 85.6% similarity with NAT which originates from traditional Japanese food natto and exhibited strong fibrinolytic enzyme activity. Furthermore, the DFE27 can both directly degrade fibrin and hydrolyze fibrin through activating plasminogen into plasmin. These unique characteristics and the sequence difference demonstrate that it is a novel fibrinolytic enzyme from *Douchi* and has wide potential applications in food or medicine for the treatment and prevention of thrombosis. Further studies should focus on the thrombolytic effects of DFE27 *in vitro* and *in vivo* and optimization of its dose in drugs or food additives.

## Supplementary information

Supplementary information

## Data Availability

The authors declared all data are available.
