# Supplementary material for: Purification and characterization of a novel, highly potent fibrinolytic enzyme from Bacillus subtilis DC27 screened from Douchi, a traditional Chinese fermented soybean food
Source: Sci Rep. 2019 Jun 25;9:9235. doi: 10.1038/s41598-019-45686-y (PMC6592948; doi:10.1038/s41598-019-45686-y)
Supplement: Supplementary file 1 — Supplementary information [file 41598_2019_45686_MOESM1_ESM.doc]

**supplementary materials**

**Journal:** Scientific Reports

**Purification and characterization of a novel, highly potent fibrinolytic enzyme from *Bacillus subtilis* DC27 screened from *Douchi*, a traditional Chinese fermented soybean food**

**Yuanliang Hu^1,2†^, Dan Yu^1†^, Zhaoting Wang^1^,** **Jianjun Hou^2^****,** **Rohit Tyagi^1^,** **Yunxiang Liang^1,3*^, Yongmei Hu^1^****^*^**

^1^ State Key Laboratory of Agricultural Microbiology, College of Life Science and Technology, Huazhong Agricultural University, Wuhan 430070, China

^2^ Hubei Key Laboratory of Edible Wild Plants Conservation＆Utilization, College of Life Sciences, Hubei Normal University, Huangshi 435002, China

^3^ Hubei Collaborative Innovation Center for Industrial Fermentation, Wuhan 430068, China

**^*^Corresponding author:** E-mail: [fa-lyx@163.com](mailto:fa-lyx@163.com) (YL), [plum73@163.com (YH)](mailto:plum73@163.com (YH))

Tel: +86 27 87281040; Fax: +86 27 87280670

State Key Laboratory of Agricultural Microbiology and College of Life Science and Technology, Huazhong Agricultural University, Wuhan 430070, P.R. China.

**^†^** These authors contributed equally to this study


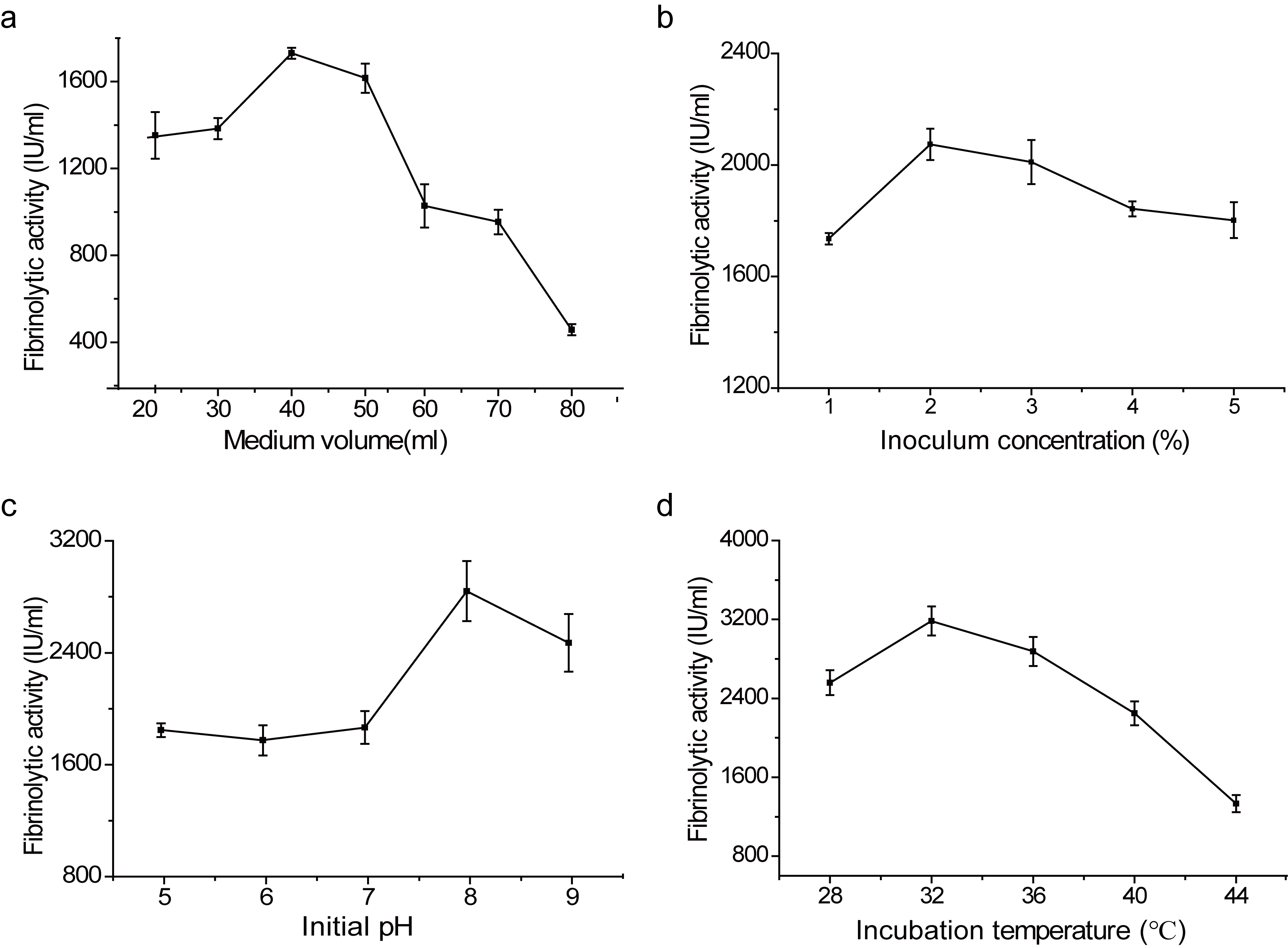


Fig. S1 Effects of culture conditions on fibrinolytic enzyme production by *B. subtilis* DC27. (a) Medium volume. (b) Inoculum concentration. (c) Initial pH. (d) Incubation temperature. All values are means ± SD of three independent experiments.

**Table S1** Biochemical tests for identification of isolate DC27

| Biochemical tests | DC27 | Biochemical tests | DC27 |
| --- | --- | --- | --- |
| Gram staining | + | Hydrolysis of starch | + |
| Catalase | + | Hydrolysis of gelatin | + |
| Voges-Proskauer test | + | Decomposition of casein | + |
| Growth in anaerobic agar | + | Lactose fermentation | + |
| Growth at 5 ℃ | + | Mannitol fermentation | + |
| Growth at 55 ℃ | - | Glucose fermentation | + |
| Growth in 7 % NaCl | + | D-xylose fermentation | + |
| Growth in 10 % NaCl | + | Citrate assimilation test | + |
| Growth in pH 6.8 | + | Propionate assimilation test | - |
| Growth in pH 5.7 | + | Indole production | - |
| Reduction of NO_3_^-^ to NO_2_^-^ | + | Phosphatase test | - |

+. Positive reaction; -. Negative reaction

**Table S2** Effects of carbon and nitrogen sources on DFE27 production

| Nutritional components | | Fibrinolytic activity (IU/ml) | Nutritional components | | Fibrinolytic activity (IU/ml) |
| --- | --- | --- | --- | --- | --- |
|  |  |  | Corn starch | Soy peptone |  |
| Carbon source | |  |  |  |  |
|  | Soluble Starch | 1164.27 ± 13.63 | 1 % | 1 % | 1184.27 ± 103.01 |
|  | Corn starch | 1427.39 ± 37.82 | 1 % | 2 % | 1291.42 ± 86.55 |
|  | Dextrin | 1109.67 ± 8.58 | 1 % | 3 % | 1272.16 ± 55.52 |
|  | Maltose | 1074.41 ± 38.84 |  |  |  |
|  | Sucrose | 614.28 ± 18.96 | 2 % | 1 % | 919.19 ± 44.54 |
|  | Glucose | 789.04 ± 13.63 | 2 % | 2 % | 1318.31 ± 29.04 |
|  | Fructose | 501.25 ± 21.20 | 2 % | 3 % | 1345.86 ± 48.71 |
| Nitrogen source | |  |  |  |  |
|  | Soy peptone | 1428.98 ± 2.86 | 3 % | 1 % | 1422.53 ± 79.32 |
|  | Peptone | 155.17 ± 6.99 | 3 % | 2 % | 1628.97 ± 70.51 |
|  | Beef extract | 1260.03 ± 57.54 | 3 % | 3 % | 1201.14 ± 67.72 |
|  | Yeast extract | 972.44 ± 19.18 |  |  |  |

All values are means ± SD (n=3).
